# Hemiparesis Revealing a Unique Neurological Hemophagocytic Lymphohistiocytosis in a Patient With Griscelli Syndrome Type 2

**DOI:** 10.7759/cureus.29159

**Published:** 2022-09-14

**Authors:** Othman Moueqqit, Ghanam Ayad, Madiha Benhachem, Abdelilah Lahmar, Hiba Ramdani, Miry Nadir, Mohammed Bensalah, Amal Bennani, Imane Kamaoui, Rachid Seddik, Noufissa Benajiba

**Affiliations:** 1 Department of General Medicine, Faculty of Medicine and Pharmacy of Oujda, Mohammed First University of Oujda, Oujda, MAR; 2 Pediatric Medicine, Centre Hospitalier Universitaire (CHU) Mohammed VI Oujda, Oujda, MAR; 3 Department of Pediatrics, Faculty of Medicine and Pharmacy of Oujda, Mohammed VI University Hospital, Mohammed First University of Oujda, Oujda, MAR; 4 Department of Medicine, Faculty of Medicine and Pharmacy of Oujda, Mohammed VI University Hospital, Mohammed First University of Oujda, Oujda, MAR; 5 Department of Pathology, Faculty of Medicine and Pharmacy of Oujda, Mohammed VI University Hospital, Mohammed First University of Oujda, Oujda, MAR; 6 Department of Hematology and Oncology, Faculty of Medicine and Pharmacy of Oujda, Mohammed VI University Hospital, Mohammed First University of Oujda, Oujda, MAR; 7 Department of Anatomopathology, Faculty of Medicine and Pharmacy of Oujda, Mohammed VI University Hospital, Mohammed First University of Oujda, Oujda, MAR; 8 Department of Radiology, Faculty of Medicine and Pharmacy of Oujda, Mohammed VI University Hospital, Mohammed First University of Oujda, Oujda, MAR; 9 Department of Hematology, Faculty of Medicine and Pharmacy of Oujda, Mohammed VI University Hospital, Mohammed First University of Oujda, Oujda, MAR; 10 Pediatric Hematology, Centre Hospitalier Universitaire (CHU) Mohammed VI Oujda, Oujda, MAR

**Keywords:** hemophagocytic lymphohistiocytosis, seizures, albinism, macrophage activation syndrome, griscelli syndrome

## Abstract

Griscelli syndrome (GS) is a rare genetic disorder that encompasses three different subtypes (GS type 1 (GS1), GS type 2 (GS2), and GS type 3 (GS3)), in which isolated neurological manifestations without immune system implications are typically seen in GS1, while neurological involvements in GS2 should be attributed to the macrophage and lymphocyte invasion of the central nervous system (CNS), under associated hemophagocytic lymphohistiocytosis (HLH). The presence of the clinical, biological, and hematologic features of HLH help explain the neurological defects that GS2 patients unusually present. In our case report, however, we attempt to highlight an uncommon presentation of GS2 involving a hemiparesis, along which we did not have any clinical or biological features of HLH. We also collect and evaluate similar published cases that feature this problem of explaining the neurological manifestations among GS2 patients.

## Introduction

Griscelli syndrome (GS) is a rare recessive autosomal disease that involves hypopigmentation of the hair and the skin, immunodeficiency with susceptibility to hemophagocytic lymphohistiocytosis (HLH), and neurological features [1]. While there are currently three subtypes described according to the genes involved in the genetic defect, only GS type 1 (GS1) is characterized by primary neurological impairments, and only GS type 2 (GS2) is described to involve immunological deficiency with associated HLH [2].

HLH usually occurs as a complication of various systemic diseases such as infections, malignancies, and autoimmune disorders, especially within the pediatric population [3]. Its clinical features include prolonged fever, hepatosplenomegaly, lymphadenopathy, hemorrhagic presentations, and sepsis-like conditions [4]. Cytopenia, coagulopathy, hypertriglyceridemia, hyperferritinemia, and a hemophagocytosis feature at myelogram are among the abnormalities of laboratory investigations [4].

Here, we report a case of a nine-month-old male with Griscelli syndrome type 2 and a previous HLH episode who presented during his hospitalization a sudden installation of a hemiparesis, without any clinical or biological features of HLH.

## Case presentation

A nine-month-old infant who is followed in our pediatric department since the age of three months for his primary immunodeficiency from Griscelli syndrome type 2 presented with a right hemiparesis affecting the face and upper and lower limbs after 21 weeks of treatment initiation.

**Figure 1:**
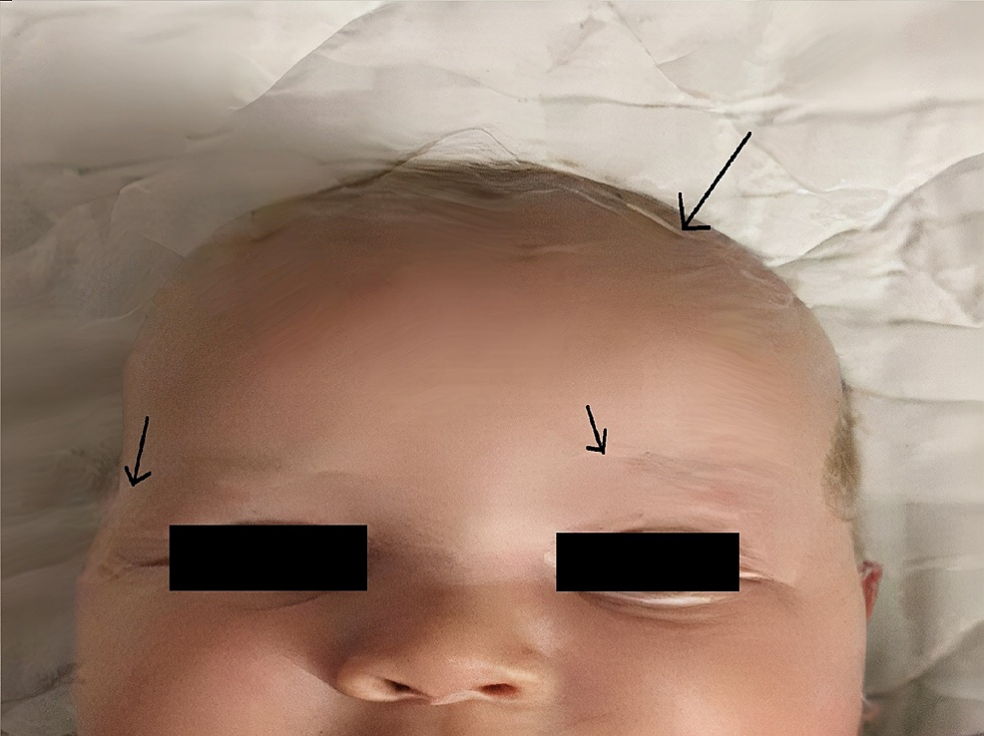
Partial albinism manifested as silvery-gray hair on the scalp and eyebrows of the patient (arrows)

The story of the infant dates back to when he was first admitted to the pediatric hematology oncology department at the age of three months for a prolonged fever for 10 days that did not respond to antipyretics, with a history of diarrhea, followed by the appearance of mucocutaneous pallor and fatiguability during breastfeeding. He also had a history of two deceased brothers, one at the age of four months and the other at the age of 16 months, with both deaths succeeding a history of prolonged fever. The infant was born out of non-consanguineous marriage, with no notion of delayed umbilical separation at birth, and received vaccination on time. No night sweating was reported, and he had no known contact with a tuberculosis patient. At his first clinical presentation, he was apyretic (37.2°C), but he had a slight skin pallor and silvery-gray hair on the scalp and eyebrows, reported as being present since birth (Figure 1). The clinical examination of the patient also found huge splenomegaly and hepatomegaly. Other than that, our patient was conscious and alert with a well-preserved motricity in four limbs.

**Figure 2:**
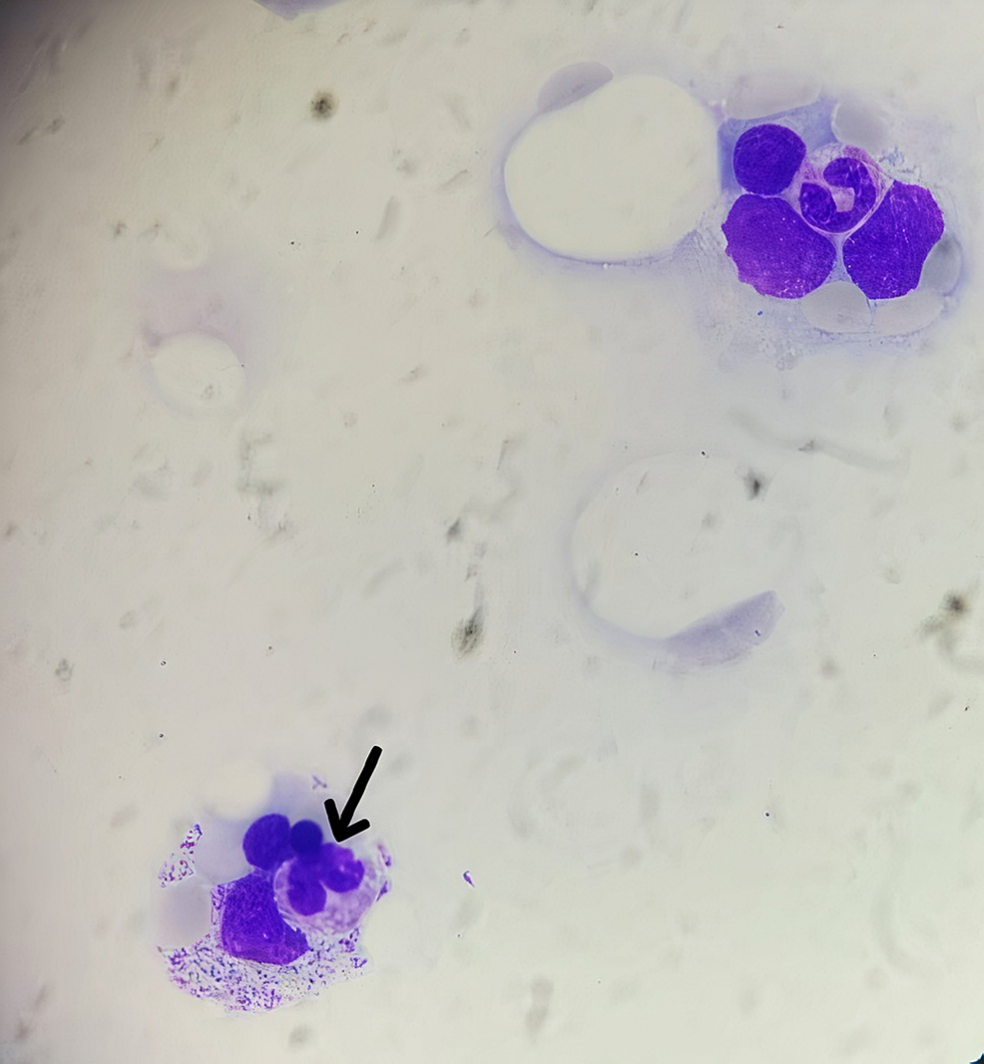
Hemophagocytosis on myelogram (arrow)

At that time (three months of age), considering the partial albinism and organomegaly found at the examination, the history of the early death of the patient’s two brothers and the anemic syndrome he presented with, and the absence of primary neurological involvements, Griscelli syndrome type 2 with associated HLH was suspected. Initial laboratory tests were initiated immediately after hospitalization (complete blood count, serum ferritin, serum triglycerides, and fibrinogen) and showed all the biological features of HLH (Table 1), along with the images of hemophagocytosis in myelogram (Figure 2).

**Table 1:** Hemophagocytic lymphohistiocytosis features in the initial laboratory results of the patient

Test	Results	Unit
Hemoglobin (Hb)	7	g/dL
Hematocrit	21.5	%
Mean corpuscular hemoglobin concentration (MCHC)	32	%
Platelet (PLT) count	53,000	Elements/mm^3^
Fibrinogen	1	g/L
Serum ferritin	496.9	µg/L
Triglyceride	6.74	g/L

**Figure 3:**
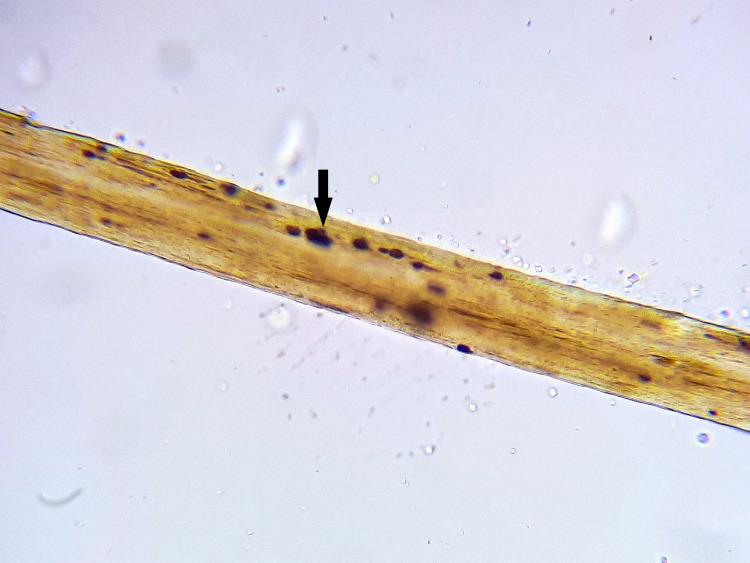
Microscopic examination of a hair shaft showing an abnormal distribution of melanin pigments, forming large pigment clumps irregularly distributed along the medullary region of the hair shaft (arrow)

During the same initial admission period, a light microscopic evaluation of the partial albinism of the hair was performed, revealing the abnormal distribution of melanin pigments, forming large pigment clumps irregularly distributed along the medullary region of the hair shaft (Figure 3). Accordingly, the diagnosis of Griscelli syndrome type 2 with associated HLH was made following the clinical and microscopic findings mentioned above, and the patient was started on the HLH-2004 etoposide-based protocol. The evolution was favorable, and the organomegaly and all the hematologic abnormalities disappeared.

After 21 weeks from the protocol initiation (at nine months of age), the infant presented with a right hemiparesis affecting the face and upper and lower limbs. The patient was apyretic at the examination and showed no signs of organomegaly or other clinical abnormalities this time. Repeated laboratory tests showed no biological signs of HLH, and his cerebrospinal fluid (CSF) test (with polymerase chain reaction (PCR)) was normal.

**Figure 4:**
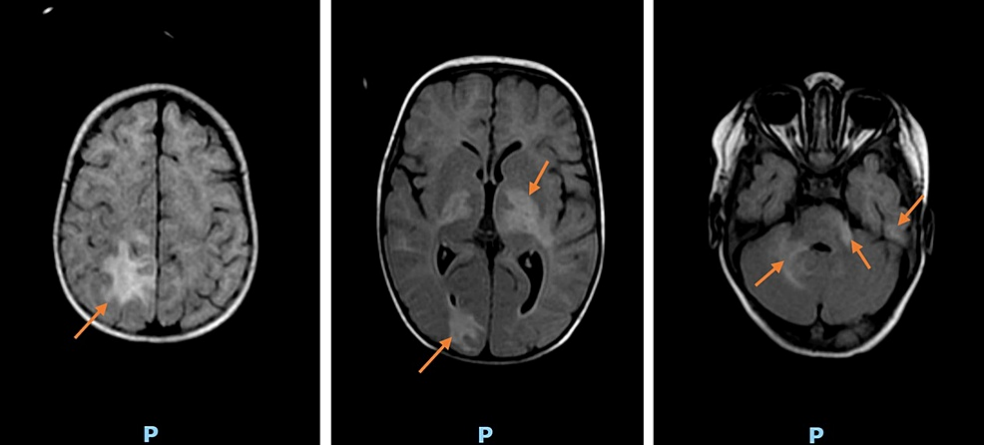
T2 fluid-attenuated inversion recovery images showing increased signal in cortical and subcortical regions of the occipital, frontal, parietal, and lentriculo-capsulo-caudal areas (arrows)

A brain magnetic resonance imaging (MRI) was performed showing T2 and T2 fluid-attenuated inversion recovery (FLAIR) increased signal in cortical and subcortical regions of the right anterior frontal lobe, right posterior parietal, left parietal, and lentriculo-capsulo-caudal region, which we could not distinguish whether they were from a local activation of his previously known HLH or presumed infectious encephalitis (Figure 4).

In light of this, the decision was to resume the HLH-2004 etoposide treatment with four intrathecal therapies per week for four weeks while rushing a wide-spectrum antibiotic treatment simultaneously. The infant was put on voriconazole, acyclovir, ciprofloxacin, and corticosteroid. Shortly after, the evolution was marked by the appearance of a bulging fontanelle and brief facial myoclonic seizures. We stopped acyclovir and the antibiotics as the septic workup was found negative, and the infant was given sodium valproate for his facial seizures. However, the hemiparesis did not resolve completely as the infant continued presenting a right upper limb paresis with an increased tone of flexor muscles in the same limb for two months. However, the decision was to go for physical rehabilitation only. Other than that, the infant continued his chemotherapy with a better evolution of his clinical state. The partial upper right limb paresis resolved progressively after six months. To date, the patient is kept under regular follow-up and reported no similar neurological implications.

## Discussion

Griscelli syndrome (GS) is a rare autosomal recessive genetic disorder, with three subtypes described according to the genes involved in this disease. The mutations in the myosin-Va (*MYO5A*) gene and the Ras-related protein Rab-27A (*RAB27A*) gene, both on chromosome 15q21, identify GS1 and GS2, respectively, while the gene encoding for melanophilin in chromosome 2q371 is responsible for GS type 3 (GS3) [5]. These mutations are associated with partial albinism, manifesting as hypopigmentation of the hair and the skin, with silvery-gray hair in all three subtypes of the disease [2]. Type 1 phenotype comes with a severe primary neurological involvement with a normal state of the immune system, and type 2 is marked by immune system deficiency and hemophagocytic lymphohistiocytosis (HLH), whereas type 3 phenotype is marked with an isolated manifestation of these symptoms [2].

Griscelli syndrome was first described in 1978 by Griscelli et al. in two unrelated patients who were described to have partial albinism, recurrent infections, and several episodes of fever [6]. Among the three described subtypes of Griscelli syndrome, GS2 is the most common and severe one, while GS3 is the least frequent and the most benign [7,8]. The diagnosis of GS may be assessed based only on the combination of clinical suspicion and the light microscopic analysis of the hair shafts [9]. However, molecular diagnosis with the detection of *RAB27A* mutation is the standard if available [9].

The differential diagnosis of this disease includes other genetic defects that share comparable clinical features, such as Chédiak-Higashi syndrome, Hermansky-Pudlak syndrome type 2, and Elejalde­ syndrome [2]. All these entities, alongside the three subtypes of GS, manifest with partial albinism [2]. Neurological symptoms can be seen in all these syndromes, except in GS3 [2], and immune system deficiencies can be seen in all of them, except in GS1, GS3, and Elejalde­ syndrome [2]. However, the presence in the blood smear of giant intracytoplasmic granules in leucocytes is unique to Chédiak-Higashi syndrome and differentiates it from the other syndromes [10]. Elejalde­ syndrome, also considered a part of GS1 by most authors [1], was linked to important ophthalmological alterations [1].

Different challenges in making the diagnosis of Griscelli syndrome have been discussed in the literature. Singh et al. have reported a case of Griscelli syndrome type 2 in a two-and-a-half-year-old male, whose diagnosis was delayed because of the attribution of his partial albinism to the very common malnutrition among infants of the same age in India, in addition to the fact that the patient came from an endemic region of Kala Azar, another disease that has similar clinical and biological manifestations to what was described in Griscelli syndrome [7]. Alqasimi et al. reported a case of a 10-year-old male whose GS2 diagnosis was delayed due to the absence of pigmentary changes, thinking it was a secondary HLH instead [11].

The neurological manifestations of Griscelli syndrome are most common in GS1. However, in many reported cases, GS2 patients presented various neurological involvements such as raised intracranial pressure, seizures, ataxia, hemiparesis, and psychomotor retardation [2,12-14], and since the *RAB27A* gene, the one responsible for GS2, is not expressed in neuronal cells, the neurological involvements reported among GS2 patients are attributed to histiocytic infiltration of the central nervous system, with the presence of most of the clinical and biological features of HLH [9]. Accordingly, cases of neurological involvements without hematologic manifestations of HLH are rarely described among GS2 patients. Our case highlights this possibility and discusses the diagnosis problem that stems from it.

Since GS2 is known for its associated primary immunodeficiency and as our patient had no organomegaly and no biological signs of HLH, to explain the hemiparesis, the bulging fontanelle, and the facial seizures, we had to include the suspicion of infectious encephalitis. Consequently, the infant was given broad-spectrum antibiotics along with acyclovir before the septic workup turned negative. This diagnosis problem that emerges from these types of local, isolated activation of the macrophages inside the central nervous system (CNS) is widely discussed among authors [12,14-16]. Similar to our case report, other cases were described and highlighted the same unusual presentation. A case of a 13-year-old female was reported by Akunuri et al. to have presented progressive ataxia due to her GS type 2, without the hematologic manifestations of macrophage hyperactivation [12]. Another unique case of an 11-year-old female was found to have presented facial palsy and hemiplegia along with immunological deficiency, but no evidence of hemophagocytosis [15]. The absence of organomegaly is also reported in a similar neurological presentation of GS2, as obstructive hydrocephalus was reported in a female without any associated organomegaly or hematologic abnormalities [14].

The exact incidence of the disease remains unknown [17]. However, according to a report by Veeramani, up to 2019, only 65 cases of GS were reported [18]. The treatment of the disease depends on the subtype. GS2 is the one with the worst prognosis, and it is usually fatal unless bone marrow transplantation (BMT) is carried out [19]. However, similar to our case, other cases reported the use of high doses of corticosteroids, immunosuppressive drugs, cyclosporine A, and etoposide as palliative therapy to delay the hyperactivation of macrophages and lymphocytes and reduce the symptoms linked to organ infiltration [19,20]. These findings explain the clinical improvement we obtained with our patient using the HLH-2004 protocol.

## Conclusions

GS2 can manifest exclusively with neurological manifestations without the more common hemophagocytic lymphohistiocytosis, causing different diagnosis problems. Multiple GS2 patients were reported to have either no hematologic manifestations of HLH or no organomegaly after they present a neurological deficit. The presence of characteristic lesions in CNS MRI along with an immunodeficiency syndrome may aid in diagnosing and distinguishing a typical GS1 from the exclusive neurological activations of HLH that follow GS2. Early diagnosis is crucial to the prognosis of GS2 patients as this form is usually fatal and requires early intervention.
